# Assessment of Antimicrobial Activity, Membrane Integrity, and Oxidative Stress of Green Synthesized Silver Nanoparticles: In Vitro and Molecular Docking Studies

**DOI:** 10.1002/open.70237

**Published:** 2026-06-02

**Authors:** Sarra Sebti, Badra Barhouchi, Samir Meskaldji, Haroun Houicha, Eyup Bagci, Hamdi Bendif, Walid Elfalleh, Stefania Garzoli

**Affiliations:** ^1^ Laboratory of Biotechnology Higher National School of Biotechnology Taoufik Khaznadar Ali Mendjeli Constantine Algeria; ^2^ Biotechnology Research Center (CRBt) Ali Mendjeli Constantine Algeria; ^3^ Pharmaceutical Sciences Research Center (CRSP) Constantine Algeria; ^4^ École Normale Supérieure de l’Enseignement Technologique (ENSET) Skikda Algeria; ^5^ Laboratoire de Physique Mathématique et Subatomique (LPMPS) Université des frères Mentouri Constantine 1 Algeria; ^6^ Laboratory of Mechanical Engineering University Mohamed Khider of Biskra Biskra Algeria; ^7^ Department of Biology Faculty of Science Firat University Elazig Turkey; ^8^ Department of Biology College of Science Imam Mohammad Ibn Saud Islamic University (IMSIU) Riyadh Saudi Arabia; ^9^ Department of Chemistry and Technologies of Drug Sapienza University Rome Italy

**Keywords:** *Acacia* *karroo*, AgNPs, antimicrobial activity, lipid peroxidation, molecular docking, oxidative stress

## Abstract

Silver nanoparticles were synthesized using *Acacia* *karroo aqueous* leaves extract for the first time, as evidenced by a characteristic UV–Vis absorption peak at 424 nm. Spherical morphologies were observed using transmission electron microscopy (TEM) and scanning electron microscopy (SEM), with mean diameters determined to be 33 nm. XRD analysis validated the crystalline nature of the nanoparticles, with characteristic peaks at 2*θ* values of 38.19°, 44.34°, 46.21°, 64.35°, 77.54°, and 81.39° corresponding to the (111), (200), (211), (220), (311), and (222) planes of face‐centered cubic silver. Synthesized AgNPs demonstrated moderate anti‐*Candida albicans* and antibacterial properties against the tested strains. Overall, AgNPs‐treated *Staphylococcus aureus* and *Escherichia coli* showed higher levels of cell leakage, protein content, and malondialdehyde concentrations than untreated cells. Molecular docking studies demonstrated strong interactions between silver and microbial proteins, with the highest binding affinity observed for protein 5NG5, followed by 5BNM and 3HUM.

## Graphical abstract

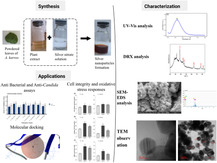

## 1 Introduction

In nanotechnology, the control and modification of nanoscale materials drive ongoing scientific progress [1]. At this scale, materials exhibit unique properties that differ significantly from those observed in their bulk counterparts [2]. Silver nanoparticles have been widely studied for their strong, well‐documented antimicrobial properties [3]. In addition, they are used in food packaging, industrial applications, the modern healthcare sector, and water treatment [4]. Therefore, developing reliable and environmentally friendly methods for synthesizing silver nanoparticles is important. Green synthesis offers significant environmental and technological advantages by reducing the use of hazardous chemicals and avoiding unfavorable synthesis conditions [4]. This approach uses biomolecules derived from plants, bacteria, and fungi that act as reducing and stabilizing agents in the process of nanoparticle synthesis [5]. Various plant species, including those of the genus *Acacia*, have been explored for the synthesis of silver nanoparticles. *Acacia* *karroo* is of particular interest due to its rich phytochemical composition. Its leaves contain significant levels of condensed tannins and phenols, while their protein and amino acid profiles compare favorably with those of other *Acacia* species [6, 7].

Previous studies investigating the mechanism of action of silver nanoparticles have consistently linked their effects to the overproduction of reactive oxygen species (ROS). This process induces protein carbonylation, lipid peroxidation, and depletion of intracellular glutathione levels [8]. Other investigations have attributed the mechanism to the release of silver ions (Ag^+^), which can bind to cellular components and disrupt essential physiological processes [9, 10]. In addition, spectroscopic analyses have demonstrated a strong interaction between silver nanoparticles and proteins, supporting their binding affinity [8]. However, the precise mechanisms underlying the biological effects of silver nanoparticles (AgNPs) remain under investigation [11, 12]. This study focuses on the synthesis of silver nanoparticles using the water‐soluble fraction of *A. karroo* and the evaluation of their antimicrobial activity. Their effects on membrane integrity will be investigated through cell and protein leakage assays, while malondialdehyde production will be measured as an indicator of oxidative stress. The study further aims to investigate interactions between silver atoms from the biosynthesized AgNPs and bacterial proteins, providing molecular‐level insight into their antibacterial mechanisms.

## 2 Methods and Materials

### 2.1 Materials

Silver nitrate and all other chemicals were of analytical grade and were provided by Sigma–Aldrich. Double‐distilled water was used throughout all experimental procedures.

### 2.2 *A. karroo* Aqueous Extract Preparation

Fresh *A. karroo* plant samples were procured in September from Skikda, Northeastern Algeria. The specimen was identified and authenticated by Mr. Mohamed Djamel Miara, botanist at Ibn Khaldoun University, Tiaret, Algeria. Healthy hand‐picked leaves were washed three times with bi‐distilled water, shade‐dried for 1 week, and ground into a fine powder. A 5 g portion of the powder were mixed with 100 mL of bi‐distilled water and heated in a water bath for 15 min. After cooling, the extract was centrifuged for 10 min at 15 000 rpm, filtered through Whatman filter paper No. 5, and the filtrate was stored at 4°C for up to 5 days.

### 2.3 *A. Karroo*‐Mediated Silver Nanoparticles (Ak‐AgNPs): Synthesis and Characterization

#### 2.3.1 Ak‐AgNPs Synthesis

Using *A.* *karroo* leaves as a dual reducing and stabilizing agent, Ak‐AgNPs were rapidly synthesized. To this end, the prepared extract and 1 mM AgNO_3_ solution were mixed in a 1:9 (v/v) ratio. The mixture was stirred in a dark water bath, with a silver nitrate control under identical conditions. Nanoparticles were isolated by centrifugation at 15 000 rpm for 10 min. The pellet was washed three times with double‐distilled water, air‐dried, and stored. Meanwhile, a purified solution was stored in the dark at room temperature for 6 months, and its stability was periodically assessed using UV–Vis analysis.

#### 2.3.2 Ak‐AgNPs Characterization

Characterization of the Ak‐AgNPs was carried out using multiple techniques. The X‐ray diffraction (XRD) pattern of the purified sample was obtained using a Bruker D2 Phaser (Bruker, Germany), equipped with Cu Kα radiation (*λ* = 0.15418 nm). Data were collected over a 2*θ* angle of 20° to 85°, with a step size of 0.02°. A dried sample portion was used to examine particle morphology and size using a Zeiss EVO MA10 scanning electron microscope (SEM) (Carl Zeiss, Germany), while elemental composition was analyzed by energy‐dispersive spectroscopy (EDS) attached to the SEM. To confirm the SEM observations, Ak‐AgNPs were further analyzed by transmission electron microscopy (TEM) (JEM‐F200, JEOL, Japan). Fourier transform infrared (FTIR) analyses were conducted using an IR Fourier spectrometer Cary 630 (Agilent Technologies, USA) with a resolution of 4 cm^−1^.

### 2.4 Evaluation of Antimicrobial Activity of Ak‐AgNPs

Overnight cultures of reference bacterial strains (*Staphylococcus aureus* ATCC 25 923, *Bacillus cereus* ATCC 10 876, *Escherichia coli* ATCC 25 922, *Klebsiella pneumoniae* ATCC 700 603, *Pseudomonas aeruginosa* ATCC 27 853, *Salmonella enteritidis* ATCC 13 076) and Mueller‐Hinton Agar (MHA) at 37°C, and clinical *Candida albicans* isolates on Sabouraud Dextrose Agar (SDA) at 30°C, were prepared in advance of the assays**.**

#### 2.4.1 Agar Well Diffusion Assay

The antibacterial and anti‐*candida* studies of Ak‐AgNPs were evaluated using the agar well diffusion method. Standardized microbial inocula (0.5 McFarland) were prepared, and 100 μL was uniformly spread onto MHA and SDA plates. Wells were aseptically introduced, and 20 μL of Ak‐AgNPs (2 mg/mL), silver nitrate (1 mM), and leaf extract (5% w/v) were added to each well. Sterile double‐distilled water and ciprofloxacin were used as negative and positive controls. Antibacterial activity and anti‐*Candida* potential were indicated by measuring inhibition zones after 24 h.

#### 2.4.2 Minimum Bactericidal/Fungicidal Concentration (MBC/MFC)

The minimum inhibitory concentration (MIC) and fungicidal concentration (MFC) of Ak‐AgNPs were assessed using the broth microdilution method in accordance with CLSI guidelines, as previously described by Jomehzadeh et al. [13]. and Ortega et al. [14]. MICs were determined using 96‐well microplates with Ak‐AgNPs in twofold serial dilutions from 2 mg/mL to 0.00012 mg/mL in MHB for bacteria and SDB for *C. albicans.* Negative and positive controls were included. MIC values were recorded visually after 24 h as the lowest concentration with no visible growth.

To determine the MBC and MFC values, 10 µl from the MIC and higher concentrations was spread onto MHA and SDA, with the lowest concentration showing no growth designated as the MBC or MFC. All assays were performed in triplicate.

### 2.5 Measurement of Cell and Protein Leakage

Cell leakage in *E. coli* and *S. aureus* was measured after incubation with Ak‐AgNPs with MIC for 20 h. Cells were removed by centrifugation at 10 000 rpm for 10 min, and the supernatant was analyzed at 260 nm for nucleic acid release. Protein concentrations were estimated at 595 nm using the Bradford method [15], with a calibration curve generated from bovine serum albumin [16].

### 2.6 Measurement of Malondialdehyde (MDA)

Lipid peroxidation was assessed as malondialdehyde (MDA) using the thiobarbituric acid assay. Untreated and Ak‐AgNPs‐treated bacterial cells (1 mL) were mixed with 30 µL of 10% sodium dodecyl sulfate (SDS) and 2 mL of 0.67% thiobarbituric acid (TBA), incubated at 95°C for 1 h, cooled, and then centrifuged at 3 500 rpm for 20 min. The absorbance of the supernatant was measured at 530 nm [16].

### 2.7 In Silico Study

To gain deeper insights into the observed biological activities, molecular docking studies were performed to evaluate the binding interactions between silver nanoparticles and bacterial target proteins. These include AcrAB−TolC protein, a membrane‐spanning efflux pump that transports multiple drugs [17], and FabH, an enzyme involved in the fatty acid biosynthesis pathway in bacteria [18] for *E. coli*, as well as the penicillin‐binding proteins, which are membrane‐bound proteins crucial for cell wall formation in *S. aureus* [19].

The crystal structure of the target proteins was retrieved from the RCSB Protein Data Bank (PDB), including the AcrAB−TolC efflux pump (PDB ID: 5NG5) and FabH (PDB ID: 5BNM) for *E. coli*, as well as 3HUM for *S. aureus*. The structures were prepared using the Molegro Virtual Docker (MVD) software suite (version 6.0) [20]. All non‐receptor heteroatoms and water molecules were systematically removed prior to docking. The MVD “Preparation Wizard” was employed to assign correct bond orders, add missing hydrogen atoms, and define protonation states and formal charges of amino acid side chains at a physiological pH of 7.4. The integrated force field parameters in MVD were then applied, incorporating a Lennard‐Jones potential for van der Waals interactions and a Coulombic potential for electrostatic interactions. A spherical search space with a radius of 15 Å was uniformly applied to the orthosteric sites of 3HUM, 5BNM, and 5NG5 to encompass the binding pocket and its adjacent regions. The molecular structure of the ligand, a neutral silver atom (Ag^0^), was generated de novo using the MVD ligand editor, with a formal charge of 0 assigned to the silver atom. The internal MVD force field parameters for silver, defining its van der Waals radius and coordination bonding potential, were subsequently applied.

Molecular docking simulations were carried out using the MVD MolDock optimizer, a heuristic search algorithm based on differential evolution. For each ligand–receptor pair, 100 independent docking runs were executed with a population size of 100 and a maximum of 2000 iterations per run. The top five poses from each run were retained for subsequent analysis. The binding affinity of each generated pose was evaluated using the MolDock Score [Grid] function, an empirical scoring function that estimates binding free energy. All poses obtained from the five runs for a given complex were consolidated and ranked according to their MolDock Scores, with the most negative value indicating the predicted most stable binding mode. The top‐ranked pose of Ag^0^ in each receptor was then subjected to detailed visual inspection to identify specific protein–ligand interactions, including potential coordination bonds with residues such as Asp, Glu, His, and Cys, as well as hydrophobic and electrostatic contacts, which were subsequently analyzed and documented.

To ensure the reliability of the molecular docking protocol, a standard re‐docking validation was performed for each target protein [20, 21]. The co‐crystallized native ligand was extracted from each PDB structure (5NG5, 5BNM, and 3HUM), prepared using the MVD Preparation Wizard, and re‐docked into its original orthosteric binding site using identical search space and optimization parameters as applied to the silver atom (Ag^0^). The root–mean‐square deviation (RMSD) between the best re‐docked pose and the experimentally determined crystallographic conformation was calculated. RMSD values of [1.91] Å (5NG5), [1.73] Å (5BNM), and [1.85] Å (3HUM) were obtained, all below the accepted 2.0 Å threshold [22, 23], confirming the accuracy and reproducibility of the docking protocol [24, 25]. The MolDock scoring function, which combines a piecewise linear potential for steric interactions with hydrogen bonding and electrostatic terms, was employed for pose evaluation [26].

### 2.8 Data Analysis

Antimicrobial activity data were analyzed by one‐way ANOVA with Tukey's post‐hoc test (*p* < 0.05) after confirming a normal distribution. Other data were compared using Student's t‐test (*p* < 0.05). Results from three independent experiments were presented as mean ± SD, and data analysis was performed in R (v4.4.2).

## 3 Results and Discussion

### 3.1 Synthesis and Characterization of Ak‐AgNPs

**FIGURE 1:**
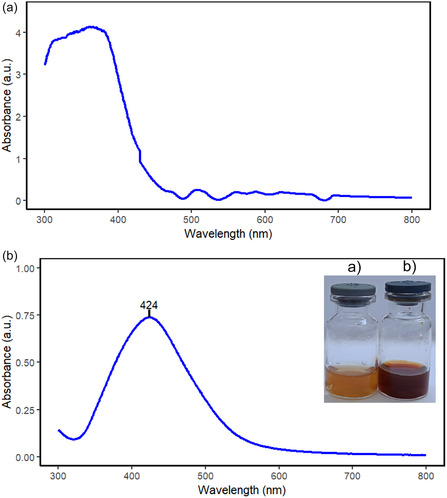
UV–Vis absorption spectrum of (a) *A.* *karroo* leaves aqueous extract (b) Ak‐AgNPs.

Mixing the aqueous extract of *A.* *karroo* leaves with silver ions in the dark produced a gradual reddish‐brown color (Figure 1) within 10 min, indicating silver nanoparticles formation. The control lacking the plant extract showed no color change, demonstrating that the biomolecules in the extract are required. The color arises from the excitation of surface Plasmon resonance (SPR), caused by the collective oscillation of free conduction electrons of AgNPs in resonance with light [27, 28, 29]. Ak‐AgNPs remained stable for 6 months without a shift in the SPR band.

#### 3.1.1 X‐ray Diffraction (XRD) Analysis

**FIGURE 2:**
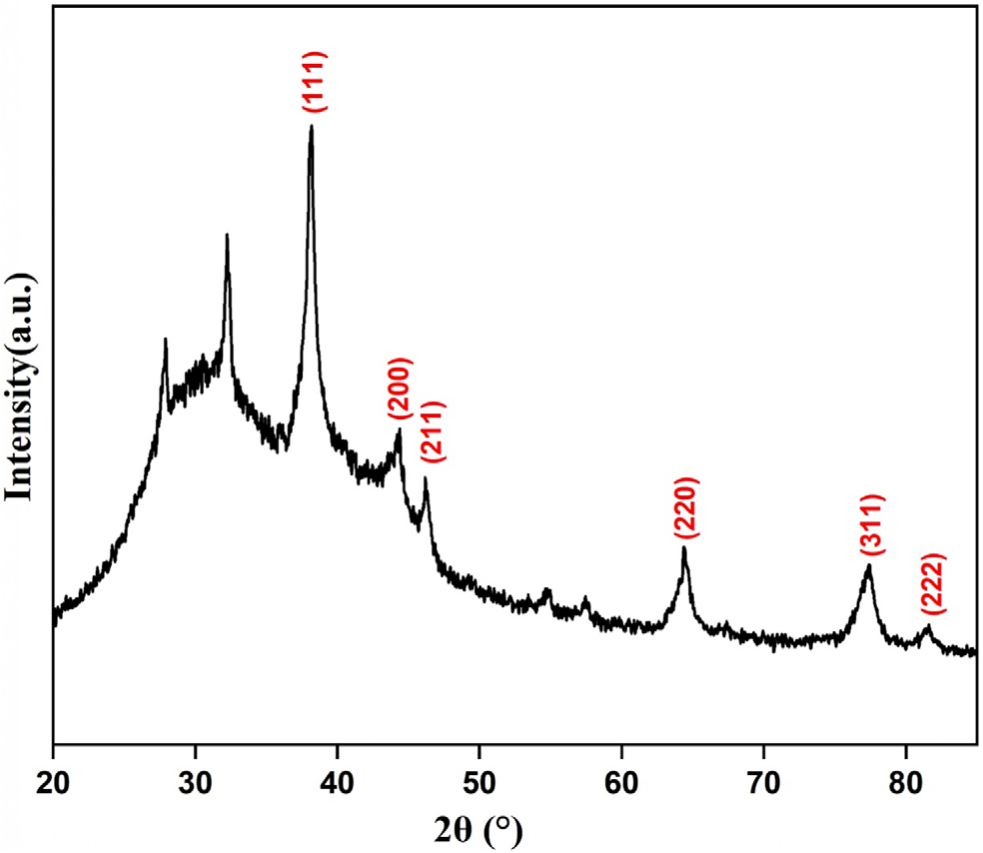
XRD pattern of Ak‐AgNPs.

XRD analysis was used to investigate the crystallinity of the Ak‐AgNPs. The XRD pattern (Figure 2) showed prominent peaks at 2θ equal to 38.19°, 44.34°, 46.21°, 64.35°, 77.54°, and 81.39°, corresponding to the (111), (200), (211), (220), (311), and (222) planes. These results were similar to previous studies [30, 31, 32]. Peaks observed at 2*θ* values of 27.85° and 32.25° likely arise from silver chloride and organic compounds [30]. The data confirm that Ak‐AgNPs have a face‐centered cubic (FCC) silver structure with a Pn‐3 m space group, a lattice constant of 4.7280 Å, and peaks consistent with JCPDS (card nu. 65−3289). The average crystallite size is 21 nm using Scherrer's equation.

#### 3.1.2 Scanning Electron Microscopy (SEM)‐EDS Analysis

**FIGURE 3:**
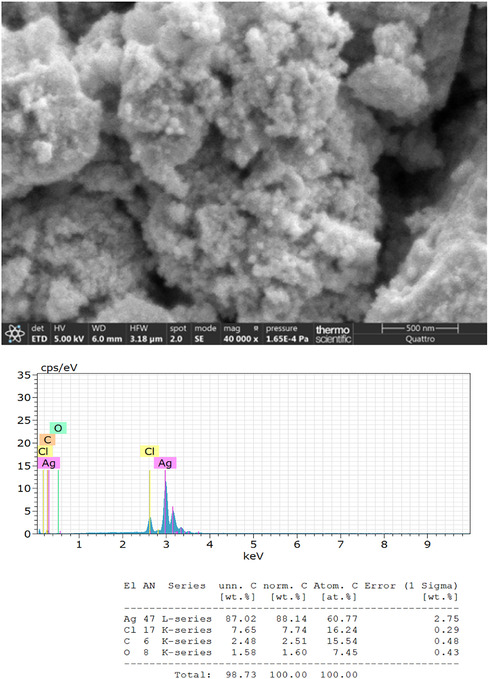
Surface morphology and elemental profile (*SEM‐EDS*) of Ak‐AgNPs.

Ak‐AgNPs characterization by scanning electron microscopy (SEM)–EDS (Figure 3) revealed a strong silver peak at 3 keV, along with weaker signals for carbon, oxygen, and trace chlorine, originating from the bio‐compounds in the extract. These biomolecules are shown to function as capping agents, stabilizing the particles [33, 34, 35]. SEM images revealed predominantly spherical particles, with occasional small clusters formed due to aggregation driven by high surface energy and van der Waals interactions [36].

#### 3.1.3 Transmission Electron Microscopy (TEM) Analysis

**FIGURE 4:**
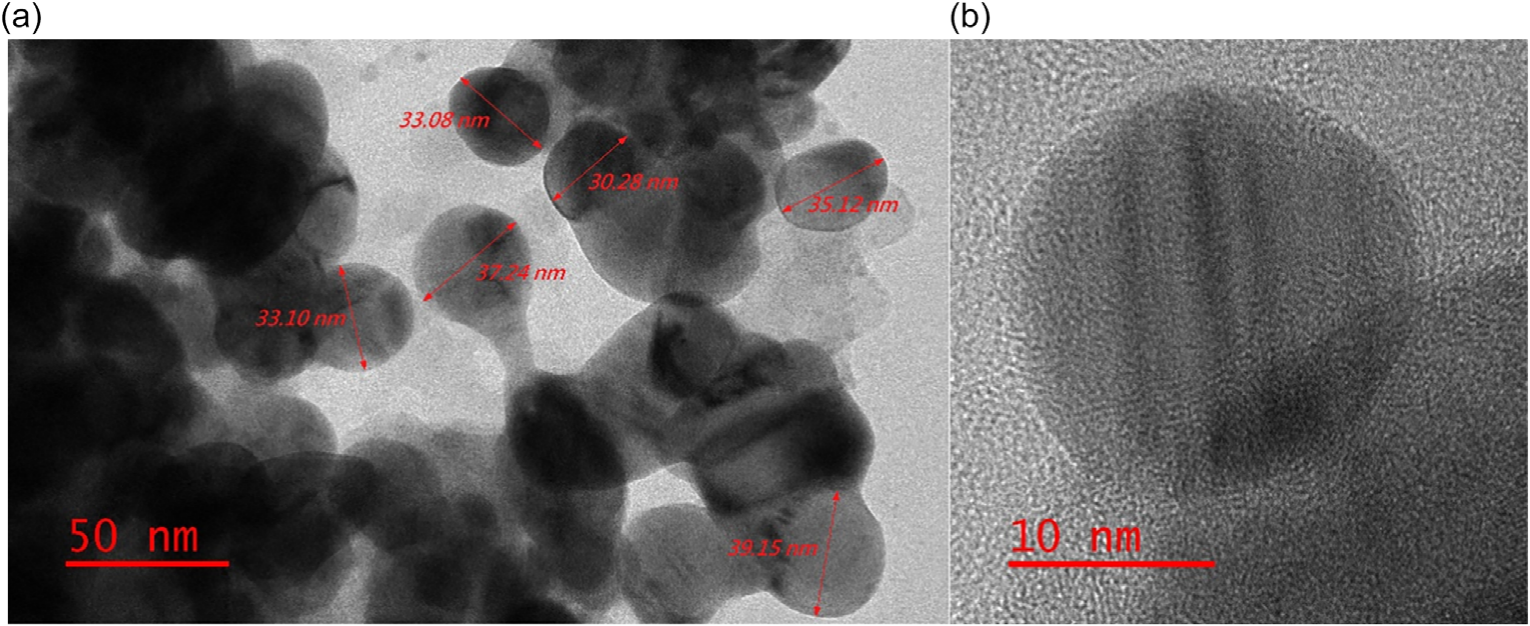
TEM images of AgNPs synthesized by *A.* *karroo* leaf extract at different scale bars (a: 50 nm; b: 10 nm).

TEM analysis was conducted to investigate the morphology and confirm the size of Ak‐AgNPs. As illustrated in Figure 4, the TEM images reveal that the nanoparticles are predominantly spherical. In this study, the average size of the obtained silver nanoparticles was estimated to be around 33 nm. These results are in good agreement with the aforementioned UV–Vis, XRD, and SEM results. The specificity of reducing and stabilizing agents is known to influence nanoparticle size, polydispersity, and surface charge [37].

#### 3.1.4 FTIR Analysis

**FIGURE 5:**
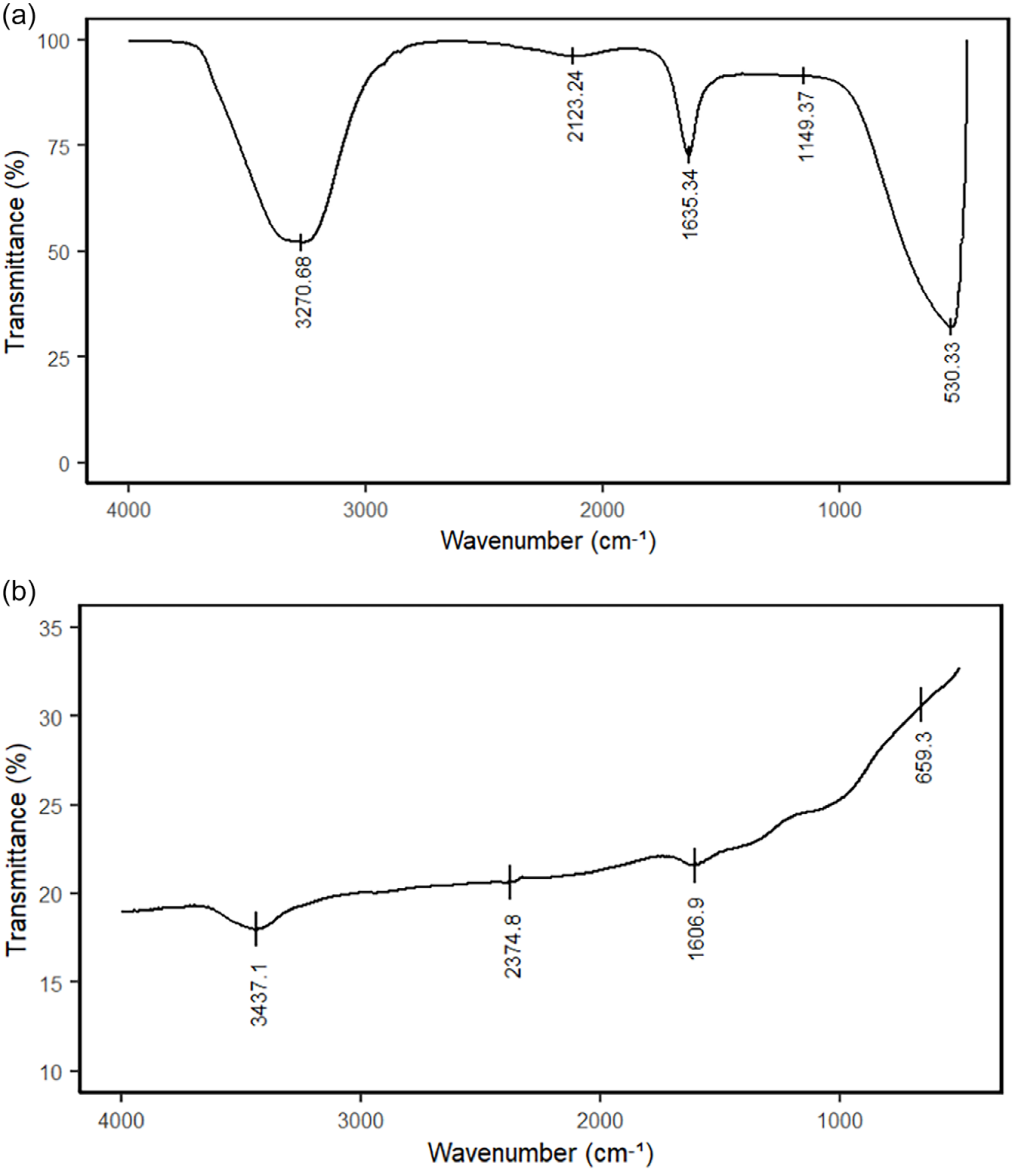
Fourier‐transform infrared spectra of (a) *A.* *karroo* leaves aqueous extract and (b) Ak‐AgNPs.

In the IR spectrum of the extract (Figure 5a), the band at 3270.68 cm^−1^ indicates –NH stretching of proteins and polypeptides, while the broad band located at 3000–3600 cm^−1^ corresponds to –OH stretching in polysaccharides [38, 39]. The band at 2114.5 cm^−1^ is attributed to —C—N bond in amide I [40]. The peak at 1635.6 cm^−1^ corresponds to C = O stretching of carboxylic groups [41], and the band at 1149.3 cm^−1^ corresponds to C–O–C stretching in glycosidic linkage of starch [42]. Also, the peak at about 530.3 cm^−1^, related cellulose skeleton vibrations, disappeared in the Ak‐AgNPs spectrum [38]. From the FTIR spectrum of Ak‐AgNPs (Figure 5b), the band at 3437.1 cm^−1^ corresponds to –OH group (hydrogen bonding) [43]. The peak at 2374.8 cm^−1^ indicates aromatic C –H groups [44]. The 1606.9 cm^−1^ band is for the C = C stretching of covalent vibration bands, which is characteristic of syringyl units in aromatic structure [45], while the band at 659.3 cm^−1^ is associated with reduced silver atoms [46].

### 3.2 Antimicrobial Activity of Ak‐AgNPs

**FIGURE 6:**
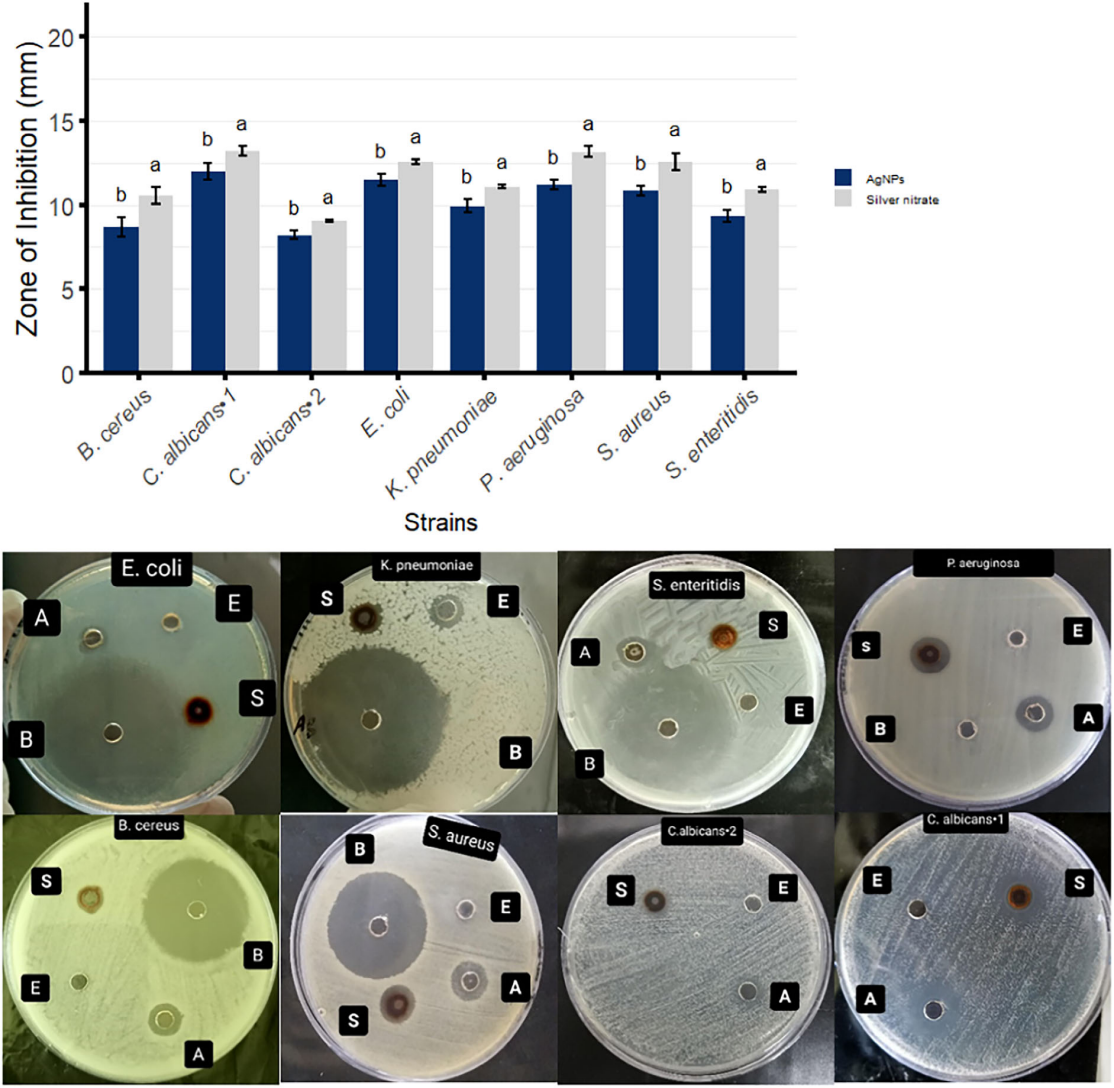
Antibacterial and anti‐*Candida* activities of Ak‐AgNPs *against tested strains.* Zones of inhibition (mm) are presented as mean ± SD (*n* = 3). No activity was observed for *A. karroo* leaves extract. Images show treatments: (A) silver nitrate, (B) antibiotic, (E) plant extract, and (S) Ak‐AgNPs.

The Ak‐AgNPs of *A.* *karroo* extract were tested for antibacterial and anti‐*Candida albicans*, using the well diffusion method. The synthesized Ak‐AgNPs showed differential effects on the tested strains (Figure 6). The highest sensitivity was observed against *E. coli* (11.5 mm) and the lowest against *B. cereus* (8.7 mm). For *C. albicans*, inhibition zones ranged from 8.2 to 12 mm, comparable to previous reports [47].

MIC and MBC or MFC of silver nanoparticles against microbial strains tested were determined using the broth microdilution method. The MIC and MBC values ranged from 500 to 1 000 µg/mL (Table 1), which are higher than those reported by Li et al. [48], who observed MIC values of 33 to 533 µg/mL against *E. coli* and *S. aureus*.

**TABLE 1:** Antimicrobial Activity of Ak‐AgNPs: MIC and MBC/MFC.

Microorganism	**Ak**‐**AgNPs**		Positive control
	MIC, µg/mL	MBC/MFC, μg/mL	MIC, µg/mL
*E. coli ATCC 25 922*	250	125	7.8
*P. aeruginosa ATCC 27 853*	250	250	—
*K. pneumoniae ATCC 700 603*	500	500	—
*S. enteritidis ATCC 13 076*	500	500	—
*S. aureus ATCC 25 923*	500	500	15.6
*B. cereus ATCC 10 876*	500	500	—
*Candida albicans•1*	1000	500	—
*Candida albicans•2*	1000	1000	—

• Laboratory of Microbiology (Clinical strain).

— not determined.

Consistent with previous studies, Ak‐AgNPs showed a strong inhibition against Gram‐negative bacteria (*E. coli* and *P. aeruginosa*) [49] likely due to their thinner peptidoglycan and specific properties, such as efflux pumps, defense mechanisms, and surface charge interactions [37, 49]. Although the exact mechanisms of antibacterial activity remain unclear [50], they are largely attributed to electrostatic forces between the positively charged nanoparticles surfaces and the negatively charged microbial cell membranes [51]. It was suggested that ions Ag^+^ released from silver nanoparticles can bind to the –SH groups of proteins, causing protein denaturation. Another proposed mechanism involves AgNPs binding to the surface proteins of fungi, leading to protein denaturation and alteration of proton pumps, which in turn increases the permeability of the protein‐lipid bilayer membrane [52]. In the referenced *E. coli*, AgNPs penetrated the cell membrane, inhibited cell division, induced leakage of cellular contents, and caused complete cell destruction [53]. This study, along with previous reports, highlights the potential of Ak‐AgNPs as anti‐*Candida* agents, with their activity reported to involve disruption of fungal membranes via ergosterol depletion [54, 55].

### 3.3 Effect of Ak‐AgNPs Treatment on Cell Leakage, Protein Quantification, and MDA Generation

**FIGURE 7:**
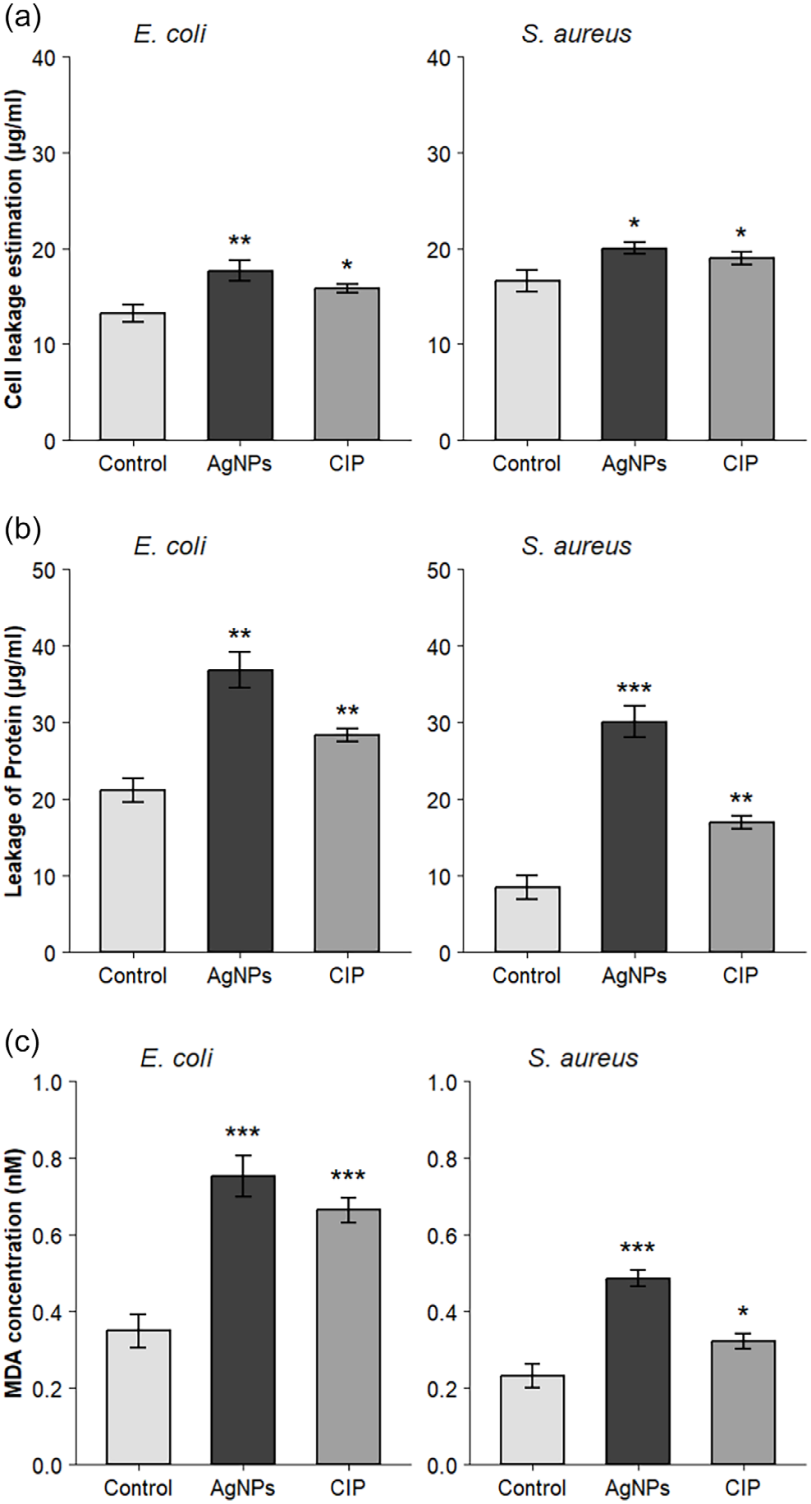
Effects of Ak‐AgNPs on (a) Cell Leakage, (b) Protein Estimation, and (c) MDA Levels. Data are presented as the mean ± SD (*n* = 3). Treated groups differed significantly from the control (Student's t‐test, *p* < 0.05).

The antibacterial effects of Ak‐AgNPs against *E. coli* and *S. aureus* were further evaluated using membrane integrity assays. Exposure to Ak‐AgNPs at their MIC increases cell leakage by 1.25‐fold in *E. coli* and 1.17‐fold in *S. aureus* (Figure 7a), protein release by 1.38‐fold in *E. coli* and 4.77‐fold in *S. aureus* (Figure 7b), and MDA levels by 2.2‐fold in *E. coli* and twofold higher in *S. aureus* (Figure 7c). These results indicate that Ak‐AgNPs exert antibacterial effects by inducing oxidative stress and lipid peroxidation, consistent with previous reports of elevated MDA levels in treated bacterial cells [12, 48, 56]. Ali et al. reported that plant‐mediated synthesized AgNPs induced the highest MDA accumulation in *K. pneumoniae* and *P. aeruginosa*, along with increased protein thiol levels in K. pneumoniae, suggesting their impact on bacterial oxidative stress and possible protein damage. Protein oxidation has also been proposed as an oxidative stress pathway induced by AgNPs. It involves the reaction of AgNPs‐induced ROS with protein molecules, leading to structural degradation and contributing to bacterial cell death [56]. Studies have also confirmed that AgNPs induce excessive production of ROS [57, 58]. Elevated ROS levels cause lipid peroxidation, increased membrane permeability, and oxidative damage to DNA, RNA, and proteins. Consequently, bacterial oxidative phosphorylation is disrupted, reducing ATP production, and AgNPs also inhibit DNA synthesis in bacterial cells. Studies have shown that ROS can upregulate ribosome regulatory factors and promote the conversion of active ribosomes into inactive dimers during the stationary phase, leading to reduced ribosomal activity and decreased protein synthesis [57]. Another proposed mechanism is the release of silver ions from AgNPs, which can bind to the bacterial cell wall and cytoplasmic membrane through electrostatic interactions and affinity toward sulfur‐containing proteins. This interaction increases membrane permeability and disrupts the integrity of the bacterial envelope. Once inside the cytoplasm, silver ions can inhibit respiratory enzymes, leading to the generation of ROS and disruption of ATP production. ROS are considered key mediators of DNA damage and membrane disruption. In addition, interactions of silver ions with sulfur‐ and phosphorus‐containing components of DNA may interfere with DNA replication and cell division, potentially leading to bacterial death. Furthermore, silver ions may induce ribosomal dysfunction in the cytoplasm, thereby inhibiting protein synthesis [59]. The findings presented herein are derived from in vitro methodologies. Detailed studies on *A.* *karroo*‐based AgNPs, especially their mechanisms of action, should be prioritized in future research. There is a need for in vivo experiments to validate their antimicrobial activity. However, their toxicological properties must be properly established to ensure their biocompatibility and safe use [60].

### 3.4 In Silico Study

Before docking the silver atom, the docking protocol was validated by re‐docking the native co‐crystallized ligands of each target protein. The resulting RMSD values (5NG5: [1.91] Å; 5BNM: [1.73] Å; 3HUM: [1.85] Å) confirmed that the MVD parameters reliably reproduce crystallographic binding poses [61, 62], thereby supporting the validity of the predicted Ag^0^–protein interactions. The use of RMSD ≤ 2.0 Å as a success criterion is well established in docking validation studies [63, 64]. The protein AcrAB−TolC, FabH from *E. coli* (PDB IDs: 5NG5 and 5BNM), and penicillin‐binding proteins from *S. aureus* (PDB ID: 3HUM), which have been identified as potential bacterial targets, were selected for the docking analysis, and the results are summarized in Table 2.

**TABLE 2:** Summary of molecular docking results for a neutral silver atom (Ag^0^) against protein target. The interaction energy (E‐Inter) represents the predicted binding affinity. Data are sorted by the best (most negative) E‐Inter value.

Protein Target	Best Pose E‐Inter, kcal/mol	**Average E‐Inter, kcal/mol** a	Number of Poses
**5NG5**	**−35.58**	**−31.38**	5
**5BNM**	−29.00	−24.15	5
**3HUM**	−24.02	−23.89	5

a Average calculated from the presented poses per protein.

For 5NG5 and 5BNM from *E. coli*, the docking binding energy between the silver atom and the target proteins was −35.58 kcal/mol and −29.00 kcal/mol, respectively. Among these, 5NG5 exhibited the most consistent binding profile, as evidenced by its highest average interaction energy of −31.38 kcal/mol. In contrast, 3HUM exhibited the weakest binding affinity (−24.02 kcal/mol). The highest binding affinity was observed for 5NG5, which interacted with Glu 238, Glu 299, and Glu 298 (Table 3).

**TABLE 3:** Molecular interaction of Ag with the active site of target proteins.

Protein	Amino acid	Bond length
5NG5	Glu 238 Glu 299 Glu 298	3.31 3.60 4.23
5BNM	His 244 Glu 302 Thr 254 Pro 243	2.52 2.00 3.70 4.06
3HUM	Asp 264 Glu 183 Arg 185 Ser 263 Thr 265	2.97 4.05 3.71 3.83 3.13

**FIGURE 8:**
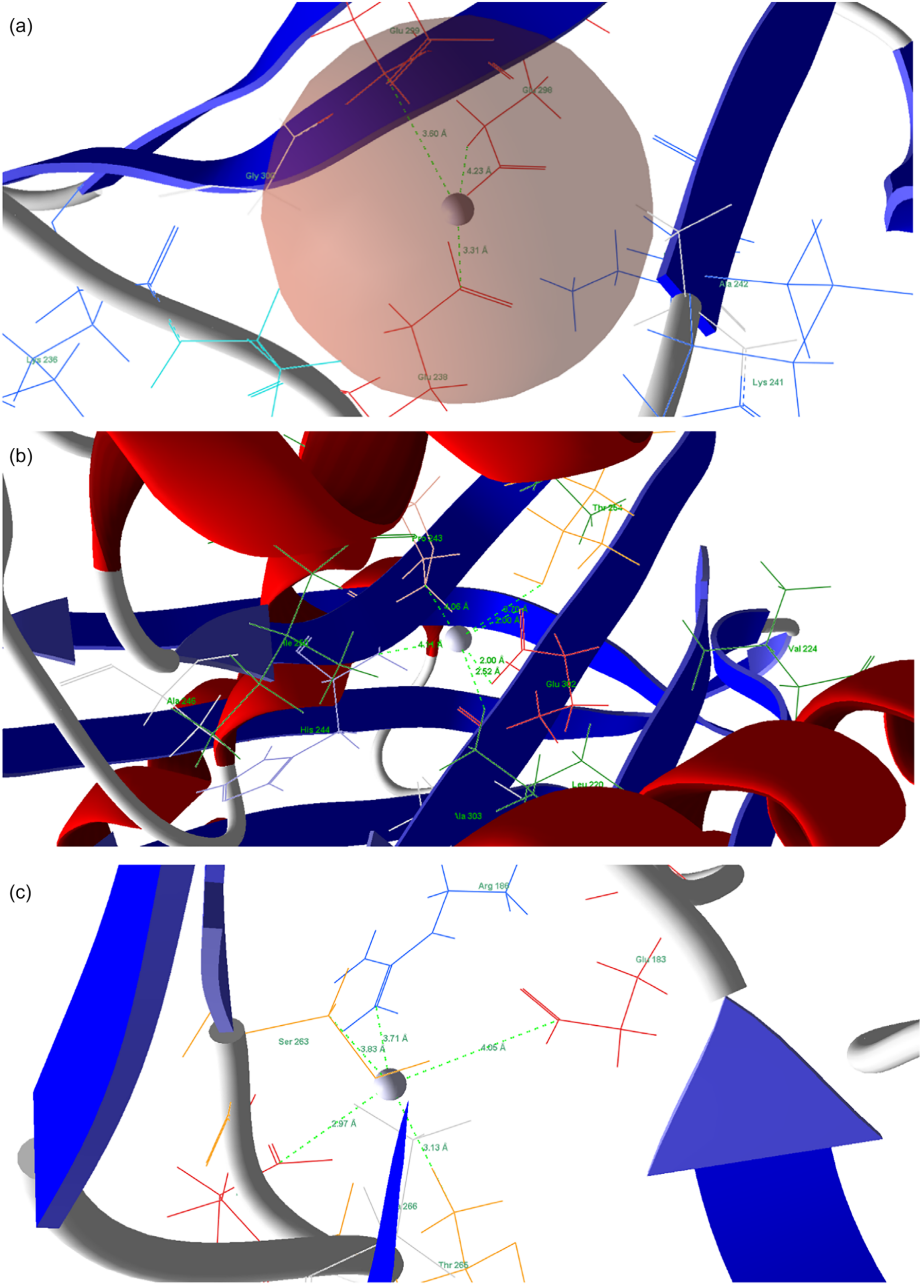
Molecular docking and interactions between silver atoms and the amino acid residues of (a) 5NG5, (b) 5BNM, and (c) 3HUM.

These sites are positioned at distances consistent with strong non‐covalent and coordination interactions, providing a structural explanation for the observed strong binding (Figure 8a).

The essential residues involved in the interaction between silver and 5BNM are His 244 and Glu 302, with interaction distances of 2.52 and 2.00 Å, respectively (Figure 8b). The 3HUM binding site for silver is primarily stabilized by a strong coordination bond with Asp 264, with additional support from a secondary polar interaction with Thr 265 (Figure 8c). Ali et al. performed molecular docking of elemental silver (Ag^0^) with quorum‐sensing proteins LasI (PDB ID: 1RO5) and LasR (PDB ID: 2UV0), as well as components of the Rhl system. The results showed that (Ag^0^) binds to key functional residues, including Asp73 of LasI (3.1 Å) and Leu159 of LasR (2.3 Å), along with His52 of RhlI (2.8 Å), Trp10 (3.1 Å), and Glu34 of RhlR (3.2 Å). These interactions suggest that silver may disrupt quorum sensing involved in biofilm formation [65]. Molecular docking analysis was performed to evaluate the interaction of elemental silver with target proteins. The results showed that Ag^0^ interacts with key functional residues of α‐amylase (Asn355 and Val358), α‐glucosidase (Gly293), and insulin (Phe24 residues). In contrast, no significant interaction was observed with glucagon, indicating selective binding toward carbohydrate‐hydrolyzing enzymes and insulin [66]. Although the binding energy of a single silver atom (Ag^0^) is lower than that of organic ligands, it can still interact favorably with amino acid residues in target proteins. These binding energies are significantly enhanced when multiple silver atoms are present in the form of AgNPs [66]. In this work, a silver atom was therefore employed as a simplified theoretical model to provide preliminary insight into potential atomic‐level interactions with key amino acid residues in target proteins.

## 4 Conclusion

The aqueous leaf extract of *A.* *karroo*, acting as reducing and stabilizing agents, enabled the synthesis of spherical, crystalline silver nanoparticles with an average diameter of 33 nm. Despite moderate antimicrobial activity against tested bacteria and *C. albicans*, Ak‐AgNPs exposure induced pronounced membrane damage in *E. coli* and *S. aureus*, evidenced by increased cell and protein leakage, as well as higher concentrations of malondialdehyde. Molecular docking indicates strong interactions of Ag with selected bacterial proteins, including the AcrAB−TolC complex and FabH in *E. coli*, alongside significant interactions with the penicillin‐binding proteins of *S. aureus*. These results highlight the potential of silver nanomaterials as advantageous alternatives; however, it should be noted that the present findings are limited to in vitro and in silico approaches. Therefore, further studies are warranted to investigate interactions with additional bacterial target proteins, and further studies are required to comprehensively understand their antimicrobial mechanisms.

## Author Contributions

**Sarra Sebti**: conceptualization, data curation, formal analysis, software, visualization, writing – original draft, writing – review & editing. **Badra Barhouchi**: supervision, resources, validation, writing – review & editing. **Samir Meskaldji**: formal analysis. **Haroun Houicha** and **Eyup Bagci**: resources. **Walid Elfalleh**: finding acquisition, project administration, formal analysis. **Stefania Garzoli**: supervision, writing – review & editing. **Hamdi Bendif**: finding acquisition, project administration, supervision, writing – review & editing.

## Open Access

Open access publishing facilitated by Universita degli Studi di Roma La Sapienza, as part of the Wiley ‐ CRUI‐CARE agreement.

## Funding

This work was supported and funded by the Deanship of Scientific Research at Imam Mohammad Ibn Saud Islamic University (IMSIU) (grant number IMSIU‐DDRSP2601).

## Conflicts of Interest

The authors declare no conflicts of interest.

## Data Availability

The data that support the findings of this study are available from the corresponding author upon reasonable request.

## References

[1] J. M. James , A. Yagoo , J. Vilvest , and A. A. A. Jessie , “Green Synthesis of Silver Nanoparticles Using *Boerhavia diffusa* Plant and the Potential of Its Antioxidant and Anticancer Efficacy,” Pharmacological Research ‐ Natural Products 6 (2025): 100142, 10.1016/j.prenap.2025.100142.

[2] M. Ohiduzzaman , M. N. I. Khan , and K. A. Khan , “Green Synthesis of Carica Papaya Mediated Silver Nanoparticles: Characterization, Antibacterial Activity, and Bioelectricity Generation for Sustainable Applications in Nanotechnology,” Journal of Molecular Structure 1317 (2024): 139141, 10.1016/j.molstruc.2024.139141.

[3] M. Baláž , N. Daneu , Ľ. Balážová , et al., “Bio‐Mechanochemical Synthesis of Silver Nanoparticles with Antibacterial Activity,” Advanced Powder Technology 28 (2017): 3307–3312, 10.1016/j.apt.2017.09.028.

[4] S. Shahzadi , S. Fatima , Q. ul Ain , Z. Shafiq , and M. R. S. A. Janjua , “A Review on Green Synthesis of Silver Nanoparticles (SNPs) Using Plant Extracts: A Multifaceted Approach in Photocatalysis, Environmental Remediation, and Biomedicine,” Rsc Advances 15 (2025): 3858–3903, 10.1039/D4RA07519F. 39917042 PMC11800103

[5] S. Singh , N. Mohammad , K. Pal , V. Dhapte‐Pawar , S. Saxena , and P. K. Khanna , “Sonochemical Synthesis of Silver Nanoparticles Using Orange Juice: Cytotoxic Behaviour and Applications,” Hybrid Advances 6 (2024): 100220, 10.1016/j.hybadv.2024.100220.

[6] C. Mapiye , M. Chimonyo , M. C. Marufu , and K. Dzama , “Utility of *Acacia karroo* for Beef Production in Southern African Smallholder Farming Systems: A Review,” Animal Feed Science and Technology 164 (2011): 135–146, 10.1016/j.anifeedsci.2011.01.006.

[7] A. Maroyi , “ *Acacia karroo*, Hayne: Ethnomedicinal Uses, Phytochemistry and Pharmacology of an Important Medicinal Plant in Southern Africa,” Asian Pacific Journal of Tropical Medicine 10 (2017): 351–360, 10.1016/j.apjtm.2017.03.021. 28552105

[8] T. Jaswal and J. Gupta , “A Review on the Toxicity of Silver Nanoparticles on Human Health,” Materials Today: Proceedings 81 (2023): 859–863, 10.1016/j.matpr.2021.04.266.

[9] Z. Xiu , Q. Zhang , H. L. Puppala , V. L. Colvin , and P. J. J. Alvarez , “Negligible Particle‐Specific Antibacterial Activity of Silver Nanoparticles,” Nano Letters 12 (2012): 4271–4275, 10.1021/nl301934w. 22765771

[10] S. Lokina , A. Stephen , V. Kaviyarasan , C. Arulvasu , and V. Narayanan , “Cytotoxicity and Antimicrobial Activities of Green Synthesized Silver Nanoparticles,” European Journal of Medicinal Chemistry 76 (2014): 256–263, 10.1016/j.ejmech.2014.02.010. 24583606

[11] N. Chatterjee , S. Pal , and P. Dhar , “Green Silver Nanoparticles from Bacteria‐ Antioxidant, Cytotoxic and Antifungal Activities,” Next Nanotechnology 6 (2024): 100089, 10.1016/j.nxnano.2024.100089.

[12] F. Fani , C. Talebpour , P. Leprohon , H. Salimnia , H. Alamdari , and M. Ouellette , “Mode of Action of Silver‐Based Perovskite against Gram‐Negative Bacteria,” Microbiology Spectrum 13 (2024): e01648–e01624, 10.1128/spectrum.01648-24. 39656007 PMC11705935

[13] N. Jomehzadeh , Z. Koolivand , E. Dahdouh , A. Akbari , A. Zahedi , and N. Chamkouri , “Investigating in‐Vitro Antimicrobial Activity, Biosynthesis, and Characterization of Silver Nanoparticles, Zinc Oxide Nanoparticles, and Silver‐Zinc Oxide Nanocomposites Using *Pistacia Atlantica Resin* ,” Materials Today. Communications 27 (2021): 102457, 10.1016/j.mtcomm.2021.102457.

[14] M. P. Ortega , L. M. López‐Marín , B. Millán‐Chiu , et al., “Polymer Mediated Synthesis of Cationic Silver Nanoparticles as an Effective Anti‐Fungal and Anti‐Biofilm Agent against *Candida* Species,” Colloid and Interface Science Communications 43 (2021): 100449, 10.1016/j.colcom.2021.100449.

[15] M. M. Bradford , “A Rapid and Sensitive Method for the Quantitation of Microgram Quantities of Protein Utilizing the Principle of Protein‐Dye Binding,” Analytical Biochemistry 72 (1976): 248–254, 10.1016/0003-2697(76)90527-3. 942051

[16] S. Perveen , N. Safdar , G. Chaudhry , and A. Yasmin , “Antibacterial Evaluation of Silver Nanoparticles Synthesized from Lychee Peel: Individual versus Antibiotic Conjugated Effects,” World Journal of Microbiology and Biotechnology 34 (2018): 118, 10.1007/s11274-018-2500-1. 30008019

[17] Z. Wang , G. Fan , C. F. Hryc , et al., “An Allosteric Transport Mechanism for the AcrAB‐TolC Multidrug Efflux Pump,” eLife 6 (2017): e24905, 10.7554/eLife.24905. 28355133 PMC5404916

[18] D. C. McKinney , C. J. Eyermann , R.‐F. Gu , et al., “Antibacterial FabH Inhibitors with Mode of Action Validated in Haemophilus Influenzae by In Vitro Resistance Mutation Mapping,” Acs Infectious Diseases 2 (2016): 456–464, 10.1021/acsinfecdis.6b00053. 27626097

[19] V. Navratna , S. Nadig , V. Sood , K. Prasad , G. Arakere , and B. Gopal , “Molecular Basis for the Role of, *Staphylococcus aureus*, Penicillin Binding Protein 4 in Antimicrobial Resistance,” Journal of Bacteriology 192 (2010): 134–144, 10.1128/jb.00822-09. 19854906 PMC2798245

[20] R. Thomsen and M. H. Christensen , “MolDock: A New Technique for High‐Accuracy Molecular Docking,” Journal of Medicinal Chemistry 49 (2006): 3315–3321, 10.1021/jm051197e. 16722650

[21] A. Castro‐Alvarez , A. M. Costa , and J. Vilarrasa , “The Performance of Several Docking Programs at Reproducing Protein–Macrolide‐Like Crystal Structures,” Molecules 22 (2017): 136, 10.3390/molecules22010136. 28106755 PMC6155922

[22] E. A. F. Martis and S. Téletchéa , “Ten Quick Tips to Perform Meaningful and Reproducible Molecular Docking Calculations,” Plos Computational Biology 21, 2025 (e1013030): 10.1371/journal.pcbi.1013030. 40344147 PMC12064015

[23] S. K. Burley , C. Bhikadiya , C. Bi , et al., “RCSB Protein Data Bank: Tools for Visualizing and Understanding Biological Macromolecules in 3D,” Protein Science 31 (2022): e4482, 10.1002/pro.4482. 36281733 PMC9667899

[24] B. J. Bender , S. Gahbauer , A. Luttens , et al., “A Practical Guide to Large‐Scale Docking,” Nature Protocols 16 (2021): 4799–4832, 10.1038/s41596-021-00597-z. 34561691 PMC8522653

[25] D. Ramírez and J. Caballero , “Is It Reliable to Take the Molecular Docking Top Scoring Position as the Best Solution without Considering Available Structural Data?,” Molecules 23 (2018): 1038, 10.3390/molecules23051038. 29710787 PMC6102569

[26] J. D. Durrant and J. A. McCammon , “Molecular Dynamics Simulations and Drug Discovery,” BMC Biology 9 (2011): 71, 10.1186/1741-7007-9-71. 22035460 PMC3203851

[27] J. Das , M. Paul Das , and P. Velusamy , “ *Sesbania grandiflora* Leaf Extract Mediated Green Synthesis of Antibacterial Silver Nanoparticles against Selected Human Pathogens,” Spectrochimica Acta. Part A, Molecular and Biomolecular Spectroscopy 104 (2013): 265–270, 10.1016/j.saa.2012.11.075. 23270884

[28] S. Arokiyaraj , M. V. Arasu , S. Vincent , et al., “Rapid Green Synthesis of Silver Nanoparticles from Chrysanthemum Indicum L and Its Antibacterial and Cytotoxic Effects: An In Vitro Study,” International Journal of Nanomedicine 9 (2014): 379–388, 10.2147/IJN.S53546. 24426782 PMC3890422

[29] M. R. Bindhu and M. Umadevi , “Surface Plasmon Resonance Optical Sensor and Antibacterial Activities of Biosynthesized Silver Nanoparticles,” Spectrochimica Acta. Part A, Molecular and Biomolecular Spectroscopy 121 (2014): 596–604, 10.1016/j.saa.2013.11.019. 24291437

[30] R. Das , P. Kumar , A. K. Singh , et al., “Green Synthesis of Silver Nanoparticles Using Trema Orientalis (L.) Extract and Evaluation of Their Antibacterial Activity,” Green Chemistry Letters and Reviews 18 (2024): 2444679, 10.1080/17518253.2024.2444679.

[31] L. David and B. Moldovan , “Green Synthesis of Biogenic Silver Nanoparticles for Efficient Catalytic Removal of Harmful Organic Dyes,” Nanomaterials 10 (2020): 202, 10.3390/nano10020202. 31991548 PMC7074911

[32] L. Wang , Y. Wu , J. Xie , S. Wu , and Z. Wu , “Characterization, Antioxidant and Antimicrobial Activities of Green Synthesized Silver Nanoparticles from, *Psidium guajava*, L. Leaf Aqueous Extracts,” Materials Science and Engineering: C 86 (2018): 1–8, 10.1016/j.msec.2018.01.003. 29525084

[33] J. Y. Song and B. S. Kim , “Rapid Biological Synthesis of Silver Nanoparticles Using Plant Leaf Extracts,” Bioprocess and Biosystems Engineering 32 (2009): 79–84, 10.1007/s00449-008-0224-6. 18438688

[34] U. B. Jagtap and V. A. Bapat , “Green Synthesis of Silver Nanoparticles Using, *Artocarpus heterophyllus*, Lam. Seed Extract and Its Antibacterial Activity,” Industrial Crops and Products 46 (2013): 132–137, 10.1016/j.indcrop.2013.01.019.

[35] J. S. Moodley , S. B. N. Krishna , K. Pillay , F. Sershen , and P. Govender , “Green Synthesis of Silver Nanoparticles from Moringa Oleifera Leaf Extracts and Its Antimicrobial Potential,” Advances in Natural Sciences: Nanoscience and Nanotechnology 9 (2018): 015011, 10.1088/2043-6254/aaabb2.

[36] G. P. Parchen , J. Volpe , M. Hospinal‐Santiani , et al., “Antibody Functionalized Thiolated Chitosan Stabilized Silver Nanoparticles as Theranostics toward Visceral Leishmaniasis,” International Journal of Biological Macromolecules 320 (2025): 145817, 10.1016/j.ijbiomac.2025.145817. 40645246

[37] W. W. Melkamu and L. T. Bitew , “Green Synthesis of Silver Nanoparticles Using Hagenia Abyssinica (Bruce) J.F. Gmel Plant Leaf Extract and Their Antibacterial and Anti‐Oxidant Activities,” Heliyon 7(2021): e08459, 10.1016/j.heliyon.2021.e08459. 34901505 PMC8642611

[38] N. Matmat , A. Abdelaziz , A. F. Tarchoun , et al., “Exploring the Effect of Ammonium Nitrate on the Thermal Stability and Decomposition Kinetics of Dual Nitrocellulose‐Nitrostarch‐Based Energetic Composites,” FirePhysChem 5 (2025): 392–402, 10.1016/j.fpc.2025.01.002.

[39] S. Dhamaratana , P. Methacanon , and S. Charoensiddhi , “Chemical Composition and *in vitro* Digestibility of Duckweed (*Wolffia globosa*) and Its Polysaccharide and Protein Fractions,” Food Chemistry Advances 6 (2025): 100867, 10.1016/j.focha.2024.100867.

[40] M. Ansari , S. Ahmed , A. Abbasi , et al., “Plant Mediated Fabrication of Silver Nanoparticles, Process Optimization, and Impact on Tomato Plant,” Scientific Reports 13 (2023): 18048, 10.1038/s41598-023-45038-x. 37872286 PMC10593853

[41] T. Wu , C. He , H. Chang , et al., “Adsorption‐Desorption Mechanisms and Migration Behavior of Fluchlordiniliprole in Four Different Soils under Varied Conditions,” Ecotoxicology and Environmental Safety 285 (2024): 117026, 10.1016/j.ecoenv.2024.117026. 39270478

[42] C. Phawachalotorn , W. Wongniramaikul , S. Kaewnoo , and A. Choodum , “Continuous‐Flow Phosphate Removal Using Cry‐Ca‐COS Monolith: Insights from Dynamic Adsorption Modeling,” Water Research X 27 (2025): 100296, 10.1016/j.wroa.2024.100296. 39811253 PMC11731986

[43] H. N. Van , L. H. T. Nguyen , N. X. D. Mai , et al., “Enhancing Docetaxel Efficacy and Reducing Toxicity Using Biodegradable Periodic Mesoporous Organosilica Nanoparticles,” Heliyon 10 (2024): e40131, 10.1016/j.heliyon.2024.e40131. 39584101 PMC11583690

[44] A. O. Faboyede , O. Bankole‐Ojo , V. F. Agbaje‐Daniel , and G. O. Adabogun , “Green Synthesis and Characterization of Silver Nanoparticles from Euphorbia Kamerunica Latex, and the Synergistic Antimicrobial Effects of Their Functionalization with Co‐Amoxiclav,” Covenant Journal of Physical and Life Sciences (2024) 12 no. 1 (2024): 1–7, https://journals.covenantuniversity.edu.ng/index.php/cjpls/article/view/4260

[45] S. Mohanaparameswari , M. Balachandramohan , P. Sasikumar , et al., “Investigation of Structural Properties and Antibacterial Activity of AgO Nanoparticle Extract from Solanum Nigrum/Mentha Leaf Extracts by Green Synthesis Method,” Green Processing and Synthesis 12 no. 1 (2023): 1–17, 10.1515/gps-2023-0080.

[46] S. Renganathan , S. Subramaniyan , N. Karunanithi , et al., “Antibacterial, Antifungal, and Antioxidant Activities of Silver Nanoparticles Biosynthesized from Bauhinia Tomentosa Linn,” Antioxidants 10 no. 12 (2021): 1–18, 10.3390/antiox10121959. PMC8749995 34943062

[47] H. A. Ghetas , N. Abdel‐Razek , M. S. Shakweer , et al., “Antimicrobial Activity of Chemically and Biologically Synthesized Silver Nanoparticles against Some Fish Pathogens,” Saudi Journal of Biological Sciences 29 (2022): 1298–1305, 10.1016/j.sjbs.2021.11.015. 35280558 PMC8913374

[48] P.‐J. Li , R.‐S. Xie , J.‐J. Pan , Y.‐Q. Jiang , and X. Liu , “Physicochemical Characterization and Antibacterial Activities of Silver Nanoparticles Prepared by Amidated Low‐Methoxyl Pectin,” RSC Advances 14 (2024): 38582–38589, 10.1039/D4RA07060G. 39650846 PMC11622035

[49] A. Y. S. Zeebaree , S. N. Mahmoud , M. G. Najm , and S. Y. Sharaf , “Bio‐Design of *Ganoderma adspersum*‐grafted Silver Nanoparticles and Assessment Their Anti‐Bacterial Efficiency,” The Microbe 9 (2025): 100573, 10.1016/j.microb.2025.100573.

[50] J. Rana , A. Sharma , J. Rana , and A. Sagar , “Phytochemical Analysis and Biogenic Synthesis of Silver Nanoparticles from Phlomis Bracteosa Royle Ex Benth. and Screening of Their Antimicrobial and Antioxidant Potential,” Micro and Nano Systems Letters 12 (2024): 29, 10.1186/s40486-024-00218-w.

[51] Hemlata , P. R. Meena , A. P. Singh , and K. K. Tejavath , “Biosynthesis of Silver Nanoparticles Using Cucumis Prophetarum Aqueous Leaf Extract and Their Antibacterial and Antiproliferative Activity Against Cancer Cell Lines,” ACS Omega 5 (2020): 5520–5528, 10.1021/acsomega.0c00155. 32201844 PMC7081640

[52] Z.‐K. Xia , Q.‐H. Ma , S.‐Y. Li , et al., “The Antifungal Effect of Silver Nanoparticles on *Trichosporon asahii* ,” Journal of Microbiology, Immunology and Infection 49 (2016): 182–188, 10.1016/j.jmii.2014.04.013. 24877597

[53] R. A. E. F. Hamouda , M. A. El‐Mongy , K. F. Eid , R. A. E. F. Hamouda , M. A. El‐Mongy , and K. F. Eid , “Antibacterial Activity of Silver Nanoparticles Using Ulva Fasciata Extracts as Reducing Agent and Sodium Dodecyl Sulfate as Stabilizer,” International Journal of Pharmacology 14, no. 3 (2018): 359–368, 10.3923/ijp.2018.359.368.

[54] H. Daoudi , A. Bouafia , S. E. Laouini , et al., “In Vitro and in Silico Study of Biosynthesized Silver Nanoparticles Using *Nigella sativa* Extract against SARS‐CoV‐2 and *Candida albicans* ,” Journal of Molecular Liquids 405 (2024): 125059, 10.1016/j.molliq.2024.125059.

[55] V. S. Radhakrishnan , M. K. R. Mudiam , M. Kumar , S. P. Dwivedi , S. P. Singh , and T. Prasad , “Silver Nanoparticles Induced Alterations in Multiple Cellular Targets, which Are Critical for Drug Susceptibilities and Pathogenicity in Fungal Pathogen (Candida Albicans),” International Journal of Nanomedicine 13 (2018): 2647–2663, 10.2147/IJN.S150648. 29760548 PMC5937493

[56] H. M. Ali , K. Karam , T. Khan , S. Wahab , S. Ullah , and M. Sadiq , “Reactive Oxygen Species Induced Oxidative Damage to DNA, Lipids, and Proteins of Antibiotic‐Resistant Bacteria by Plant‐Based Silver Nanoparticles,” 3 Biotech 13 (2023): 414, 10.1007/s13205-023-03835-1. PMC10665289 38009163

[57] S. Liao , Y. Zhang , X. Pan , et al., “Antibacterial Activity and Mechanism of Silver Nanoparticles against Multidrug‐Resistant Pseudomonas Aeruginosa,” International Journal of Nanomedicine 14 (2019): 1469–1487, 10.2147/IJN.S191340. 30880959 PMC6396885

[58] B. Liu , D. Liu , T. Chen , et al., “ITRAQ‐Based Quantitative Proteomic Analysis of the Antibacterial Mechanism of Silver Nanoparticles against Multidrug‐Resistant Streptococcus Suis,” Frontiers in Microbiology 14 (2023): 10.3389/fmicb.2023.1293363. PMC10684948 38033593

[59] P. R. More , S. Pandit , A. D. Filippis , G. Franci , I. Mijakovic , and M. Galdiero , “Silver Nanoparticles: Bactericidal and Mechanistic Approach against Drug Resistant Pathogens,” Microorganisms 11 no. 12 (2023): 1‐27, 10.3390/microorganisms11020369. PMC9961011 36838334

[60] S. Budhathoki , N. Chaudhary , B. Guragain , D. Baral , J. Adhikari , and N. K. Chaudhary , “Green Synthesis of Silver Nanoparticles from Brassaiopsis Hainla Extract for the Evaluation of Antibacterial and Anticorrosion Properties,” Heliyon 10 (2024): e35642, 10.1016/j.heliyon.2024.e35642. 39170326 PMC11336820

[61] G. M. Morris , R. Huey , W. Lindstrom , et al., “AutoDock4 and AutoDockTools4: Automated Docking with Selective Receptor Flexibility,” Journal of Computational Chemistry 30 (2009): 2785–2791, 10.1002/jcc.21256. 19399780 PMC2760638

[62] S. Dallakyan , A. J. Olson , J. E. Hempel , and C. H. Williams ,“ Small‐Molecule Library Screening by Docking with PyRx,“ in: Chemical Biology: Methods and Protocols, ed. J. E. Hempel , C. H. Williams , and C. C. Hong (Springer, 2015), 243–250, 10.1007/978-1-4939-2269-7_19. 25618350

[63] G. Sciortino , E. Garribba , and J.‐D. Maréchal , “Validation and Applications of Protein–Ligand Docking Approaches Improved for Metalloligands with Multiple Vacant Sites,” Inorganic Chemistry 58 (2019): 294–306, 10.1021/acs.inorgchem.8b02374. 30475597

[64] A. A. Buglak , R. R. Ramazanov , and A. I. Kononov , “Silver Cluster–amino Acid Interactions: A Quantum‐Chemical Study,” Amino Acids 51 (2019): 855–864, 10.1007/s00726-019-02728-z. 30900086

[65] S. G. Ali , M. A. Ansari , Q. M. Sajid Jamal , et al., “Antiquorum Sensing Activity of Silver Nanoparticles in P. Aeruginosa: An in Silico Study,” In Silico Pharmacology 5 (2017): 12, 10.1007/s40203-017-0031-3. 29098138 PMC5651536

[66] D. Jini , S. Sharmila , A. Anitha , M. Pandian , and R. M. H. Rajapaksha , “In Vitro and in Silico Studies of Silver Nanoparticles (AgNPs) from Allium Sativum against Diabetes,” Scientific Reports 12 (2022): 22109, 10.1038/s41598-022-24818-x. 36543812 PMC9772310

